# Relation of Subclinical Ketosis of Dairy Cows with Locomotion Behaviour and Ambient Temperature

**DOI:** 10.3390/ani10122311

**Published:** 2020-12-07

**Authors:** Ramūnas Antanaitis, Vida Juozaitienė, Mindaugas Televičius, Dovilė Malašauskienė, Mingaudas Urbutis, Walter Baumgartner

**Affiliations:** 1 Large Animal Clinic, Veterinary Academy, Lithuanian University of Health Sciences, Tilžės 18, LT-47181 Kaunas, Lithuania; mindaugas.televicius@lsmuni.lt (M.T.); dovile.malasauskiene@lsmuni.lt (D.M.); mingaudas.urbutis@lsmuni.lt (M.U.); 2 Department of Animal Breeding, Veterinary Academy, Lithuanian University of Health Sciences, Tilžės 18, LT-47181 Kaunas, Lithuania; vida.juozaitiene@lsmuni.lt; 3 University Clinic for Ruminants, University of Veterinary Medicine, Veterinaerplatz 1, A-1210 Vienna, Austria; walter.baumgartner@vetmeduni.ac.at

**Keywords:** sensors, innovation in animal farming, smart farming

## Abstract

**Simple Summary:**

The use of innovative tools and the registration of new biomarkers can help with identification of certain diseases in fresh dairy cows earlier and more accurately, thus improving the quality of treatment and reducing the losses incurred. One of the most often diagnosed diseases of postpartum cows is subclinical ketosis. According to our knowledge there exists limited information about how subclinical ketosis is related to locomotion behaviour (walking activity, feeding time with head position down, feeding time with head position up, change between activities) and average, minimal and maximal ambient temperature. We hypothesized that continuous maximal monitoring of cow locomotion behaviour (in combination with measuring the ambient temperature) could identify cows with subclinical ketosis. In addition, we hoped that changes of the above-mentioned parameters prior to clear clinical signs of subclinical ketosis would aid in earlier detection of the disease.

**Abstract:**

Rumination time, chewing time and drinking time are indicators that can be assessed in case of cow disease. In this research, two groups of cows were formed: cows with subclinical ketosis (SCK; *n* = 10) and healthy cows (HG; *n* = 10). Behaviour such as walking activity, feeding time with head position up, feeding time with head position down, change of activity and average, minimal and maximal ambient temperature of cows were recorded by the RumiWatch noseband system (RWS; RumiWatch System, Itin+Hoch GmbH, Liestal, Switzerland). The RWS comprises a noseband halter with a built-in pressure sensor and a liquid-filled pressure tube. Data from each studied cow were recorded for 420 h. According to the results of our study, it was determined that cows diagnosed with subclinical ketosis showed a tendency to change their activity more frequently. Our data indicates that minimal and maximal ambient temperatures are related with SCK.

## 1. Introduction

In several countries, various devices have been described for the automatic monitoring of physiological and pathological characteristic values in dairy cows [1,2,3].

Sensor-generated data can be used alone or with traditional health-monitoring protocols to identify cows with health disorders [1,4]. Furthermore, continuous monitoring of behaviour and physiological parameters may allow for the detection of subtle changes before evident clinical signs appear. Earlier disease detection may benefit cows by preventing progression and improving response to treatment [5]. Health disorders in the early postpartum period affect a substantial proportion of lactating dairy cows, with negative results for their health, welfare, and performance [6]. McArt et al. [7] reported an average cumulative subclinical ketosis (SCK) incidence of 43% among cows tested thrice weekly from 3 to 16 days in milk (DIM), with the peak incidence at 5 DIM. The current challenge for producers is identifying SCK at an early stage [8]. Additional tests to confirm the presence of subclinical disorders or underlying predisposing factors for clinical diseases may facilitate decision-making [7,9]. Future research is warranted for establishing criteria for differentiating and treating specific health disorders based on the information provided by the automatic health monitoring system (AHMS) [5]. For example, retained placenta, mastitis, displaced abomasum, metritis or lameness could have influenced the walking activity behaviour of the animals [7,10,11]. In cows with SCK, the walking activity was generally lower than that of healthy cows [12]. According to our knowledge, limited research has been done to evaluate the relationship between subclinical ketosis and changes in locomotion behaviour (time spent with the head up during feeding, time spent with head down during feeding, walking activity and frequency of switching between activities) along with the average, maximal and minimal ambient temperatures. Changes in behaviour may be a result of cows, management, or physiological factors [13].

It has been hypothesized that continuous monitoring of cow movement behaviour would lead to identification of cows with SCK. Additionally, we expected that changes in these parameters before evident clinical signs of SCK would result in earlier identification of disease. Accordingly, the aim of the current study was to determine the relation between subclinical ketosis, locomotion behaviour (feeding time with head positioned upwards, feeding time with head positioned downwards, frequency of switching between activities, and walking activity), and ambient temperature (average, minimal and maximal temperature).

## 2. Materials and Methods

### 2.1. Location, Animals and Experimental Design

The study was performed in the Southern part of Lithuania at (46°45′59.0″ N, 7°6′17.2″ E) between 1 June and 25 August 2020. Prior to selection of the cows, all possible cow candidates underwent a thorough clinical examination. Out of 120 Lithuanian Black and White breed cows, twenty were selected according to the following criteria: clinically healthy, 30 days postpartum, in their second or higher lactation (on average 3.0 ± 0.13 lactations). The cows were controlled for 18 days, from 30 to 48 days in milk. The system monitored the cows constantly 24 h per day for 17.5 days. On the day of diagnosis (day 0), data were recorded shorter, only for 12 h. A total of 420 h of data was recorded for each animal in the study. To determine the health status of the animal, every day at 9:00 a.m. a clinical examination was performed (by the same veterinarian) and the data from automatic milking systems concerning milk fat-protein ratio and blood beta-hydroxybutyrate (BHB) was recorded.

The cows were housed in a ventilated free-stall barn and were receiving total mixed ration (TMR), balanced to meet their physiological needs, throughout the year at a certain fixed time, at 06:00 a.m. and 06:00 p.m.

Diet components were formulated accordingly to meet or exceed the requirements of a 550 kg Holstein cow producing 35 +/−0.5 kg/day. Composition of ration: dry matter (DM) (%) 48.8; non fibre carbohydrates (% of DM) 38.7; neutral detergent fibre (% of DM) 28.2; acid detergent fibre (% of DM) 19.8; crude protein (% of DM) 15.8; net energy for lactation (Mcal/kg) 1.6. The average weight of the cows was 550 kg +/−45 kg. In 2019, the average energy corrected milk yield (4.2% fat, 3.5% protein) at this farm was 9500 kg per cow and per year.

The cows were milked with Lely Astronaut^®^ (Maassluis, The Netherlands) A3 milking robots with free traffic. To be able to motivate the cows to visit the robot, a minimum of 2 kg/day of concentrates were offered to them during milking in the robot.

### 2.2. Determining Health Status

For the current study, out of a total of 120 dairy cows according to the results of the general clinical examination 20 of the cows were selected and grouped: ten of these were diagnosed with subclinical ketosis (SCK), and ten were clinically healthy cows. Automatic milking system was used to register daily milk fat-protein ratio (F/P).

#### 2.2.1. Subclinical Ketosis Group (SCK; *n* = 10)

During the selection via clinical examination period, ten of the cows were classified into SCK group, since at least one beta-hydroxybutyrate (BHB) reading was ≥1.2 mmol/L. Milk fat-protein ratio (F/P) for that group of cows was registered as >1.2. Cows in this group showed no clinical signs of other post-partum diseases (metritis, mastitis, lameness, indigestion, displaced abomasum) and the average rectal temperature was 38.8 °C, rumen motility was five to six times per three minutes.

#### 2.2.2. Healthy Group (HG; *n* = 10)

The animals in this group presented no clinical signs of any disease after calving. Cows were classified into this group when all BHB readings (during their clinical examination) were at <1.2 mmol/L. The average milk F/P for this group of cows was at 1.2.

### 2.3. Measurements

The RWS (RumiWatch System; Itin+Hoch GmbH, Liestal, Switzerland) consists of a noseband halter with a liquid-filled pressure tube connected to a pressure sensor. The pressure sensor produces a pressure signal which is sent to a data logger placed in a secure plastic box on the same halter. An acceleration sensor for detection of triaxial head movements, and a durable memory cardholder are incorporated as well. The accelerations and pressure measurements are saved as binary files at a frequency of 10 Hz. A wireless data transmitter connects between the halter and the software RumiWatch Manager and makes live data acquisition possible. Simple algorithms, implemented in the RWC software, process the customised classification of behavioural characteristics from 10 Hz pressure data in different selectable time summaries. As the consolidation of 10 Hz pressure data in 1-min time summaries only facilitates a binary output of the behavioural characteristics of drinking, rumination, eating and other activity, executed within the respective minute or not, further behavioural parameters are convertible from raw pressure data in 1-h time summaries (Table 1). The classification principles of the algorithms are based on the identification of distinct pressure peak profiles, shaped by jaw movements, which are capable of being differentiated between the behavioural characteristics [14]. Ambient temperature was measured by RumiWatch System (Itin+Hoch GmbH, Liestal, Switzerland) with their patented sensor.

Plasma ketone body levels were found by using the Medi Sense and Free Style Optium H systems (Abbott, Maidenhead, UK) by taking a sampling of capillary blood from the ear. All samples were taken during clinical examination. Milk F/P was registered by Lely Astronaut^®^ A3 milking robots.

### 2.4. Data Analysis and Statistics

The SPSS 20.0 (SPSS Inc., Chicago, IL, USA) program package was used to perform the statistical analysis of RumiWatch data. With the use of descriptive statistics, normal distributions of variables were calculated using the Kolmogorov–Smirnov test. The results were stated as the mean ± standard error of the mean (M ± S.E.M). Student’s *t*-test was used to analyse the differences in the mean values of normally distributed variables between SCK and HG. A probability <0.05 was considered significant (*p* < 0.05).

The statistical relationship between day (from −17th to day 0 (day of SCK diagnosis) of the experiment, an independent variable, and RumiWatch, a dependant variable, was determined with the use of linear regression equation.

The research was carried out in accordance to the provisions of the Law on Animal Welfare and Protection of the Republic of Lithuania and regards the European Convention for the protection of vertebrate animals used for experimental and other scientific purposes (Official Journal 2007, No. 49) (1883, No. 49-1884). The study approval number was PK016965.

## 3. Results and Discussion

Automated health-monitoring systems that use activity are efficient tools for the identification of cows with metabolic and digestive disorders in dairy farms [5]. The timely detection of cows with health disorders during the early postpartum period requires consistent implementation of a comprehensive health-monitoring program [5]. Detecting a health disorder at an early stage and before the manifestation of clear clinical signs may benefit cows by improving overall treatment response and reducing the negative long-term consequences of disease on overall cow health and performance [15]. In the near future, more elaborate machine learning approaches will be used to deal with early detection of SCK.

Statistically significant differences between groups for MinT, MaxT and changes in cow activity were found (Table 2). SCK cows showed lower average values for the following parameters: MinT (1.16 times, *p* < 0.003) and MaxT (1.10 times; *p* = 0.001). However, the arithmetic mean of HG cows was lower for change of activity (1.53 times; *p* < 0.001) (Table 2).

**Figure 1:**
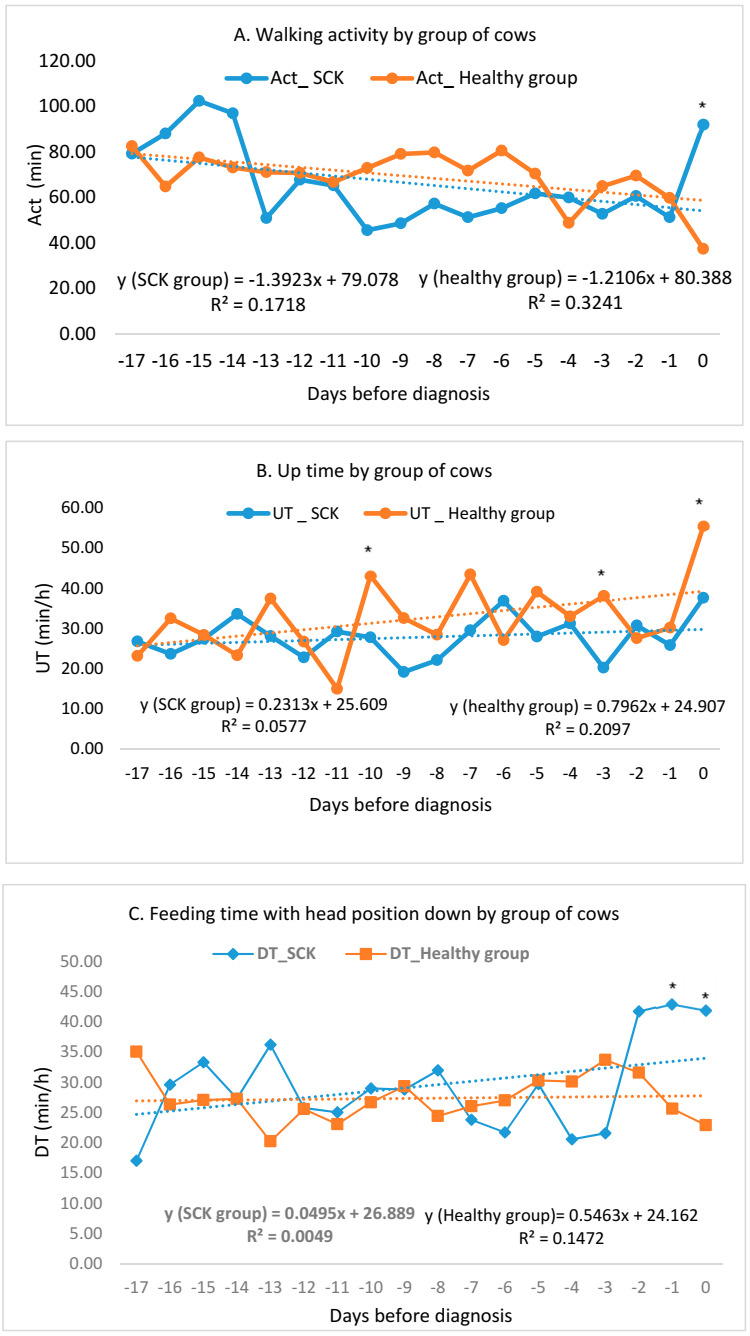

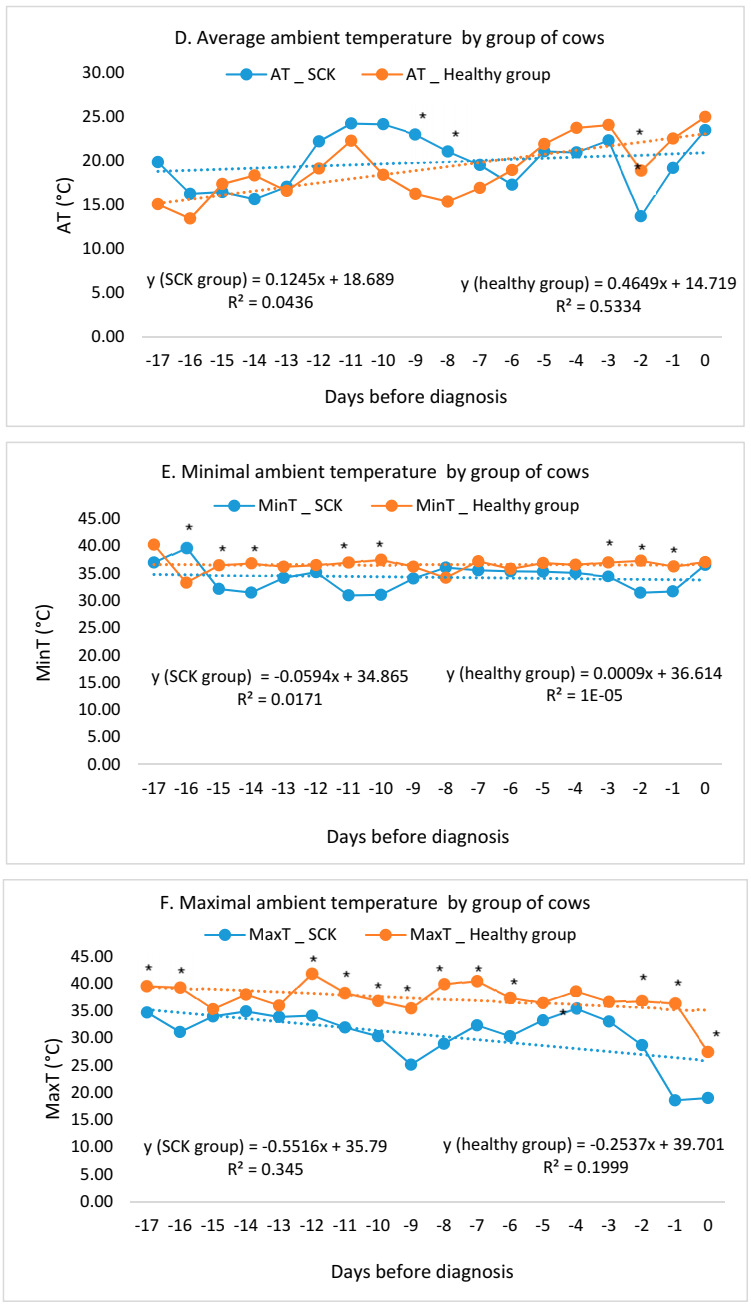

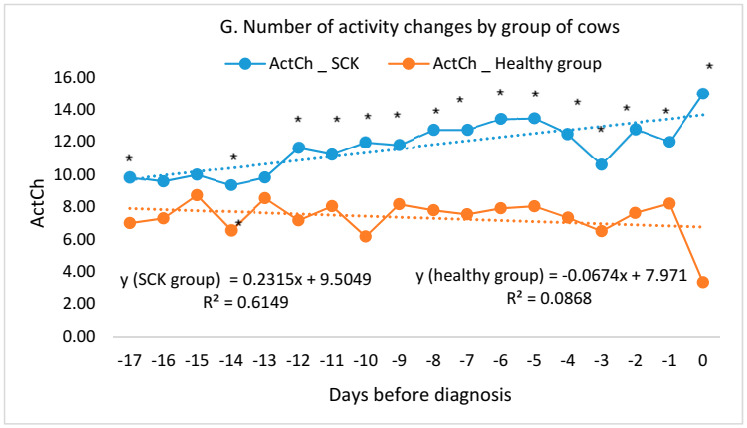
Changes in “RumiWatch” indicators during the experiment. * = significant differences (*p* < 0.05).

On the day of SCK diagnosis, up time (47.53 %, *p* < 0.001), average temperature (6.38%, *p* = 0.032) and maximal temperature (44.89%, *p* = 0.032) were significantly higher. Activity (59.42%, *p* < 0.001), down time (45.40%, *p* < 0.001) and activity change (77.78%, *p* < 0.001) were lower in HG (Figure 1).

Sturm et al. 2020 [16] reported that cows at risk for SKC are slower, i.e., fewer moves compared to healthy cows. Long lying times and a reduction in activity (i.e., paresis) are common sickness behaviours and are clinical symptoms of milk fever [17]. Lying is an important component of cow comfort and an indicator of welfare [18]. In housed systems, lying behaviour has recently been recognized as an early indicator of health problems [19] and is also of interest in grazing systems for the improved management of individual dairy cows [20]. Quantitative research that focuses on defining changes in the behaviour of healthy cows is an important consideration when using behaviour as an indicator of illness or welfare [21,22]. A study evaluating the post calving lying behaviour of a group of grazing cows highlighted differences in daily lying times when compared with lying times reported in housed cows, and it was speculated that this could be due to external factors such as feed accessibility and time spent walking to and from the milking parlour [23].

Changes in the observed indicators during experimental days are described in Figure 1. The analysis showed that the change of the following indicators maximal ambient temperature (*p* = 0.01) and activity change (*p* < 0.001) during the experiment for SCK cows can be described by a linear regression equation with a statistically reliable regression coefficient, while in HG cows, a statistically reliable regression was found for only four indicators—walking activity (*p* = 0.014) and average ambient temperature (*p* = 0.001).

Farm-specific factors such as time spent waiting to be milked [24], wintering system [25], weather [13], and other management factors need to be considered when comparing behaviour measurements from different farms or groups of animals. In housed cows, competition for space to lie down in free stables could limit the lying behaviour of some cows [26], whereas in dairy cows on pasture, competition to lie down may be less likely to disrupt normal lying behaviour due to higher space availability [27]. Ito et al. [26] reported a difference in the range of means for individual cows of 15.3 h/d and 27 no./d for daily lying time and 342 min/bout for the mean lying behaviour duration among individual housed cows.

Changes to an animal’s physiological state influence their behaviour [28]. Changes in the feeding behaviour show promise as parameters for early identification of animals that will develop metabolic or infectious diseases down the line [13,29]. The increase in step count may also be due to cows walking in search of a place to calve or seeking isolation from the herd [30]. Daily lying time declined steadily during the post calving period until d 34, in agreement with Maselyne et al. [21]. In studies under confinement housing, higher energy requirements increasing time spent eating have been attributed to less time lying [31,32] and increasing amounts of milk in the udder leading to discomfort has been attributed as a possible cause for the reduction in lying time [33]. Disease could also be a factor contributing to change in behaviour. Cows with SCK are in a state of excessive negative energy balance, as ketone bodies concentration in the blood rises while the availability of glucose is low [34]. Thus, it could be hypothesized that cows with SCK may spend more time lying down to conserve their already low energy resources. Increased lying time in sick cows could be explained by the animal trying to conserve energy for the upcoming recovery period, thus becoming less active [35].

Rutherford et al. [36] showed that cows suffering from subclinical ketosis exhibited lower peak activity and a shorter duration in activity clusters associated with first oestrus and first insemination post-partum compared with healthy cows. That may reduce the efficiency of detecting oestrus by automated surveillance systems. Motion activity is not usually used to evaluate the metabolism of animals, only a few studies indicate that it can be used as a parameter for early detection of subclinical ketosis. The motion activity could be affected by other cow diseases (mastitis, metritis, etc.) [37,38]. According to Poulopoulou et al. [39], high correlations were confirmed for the activities feeding, ruminating, standing and lying. Significantly reduced motion activity was found in ketotic animals compared to healthy animals [12]. Promising future applications include the early detection of lameness which is associated with alterations in walking activity [40], or metabolic diseases, which can be predicted by changes in feeding and ruminating behaviours [5]. Recent studies showed that subclinical and clinical diseases are associated with distinct animal behaviours, e.g., changes in rumination, as well as with standing and lying durations, respectively [5].

Higher temperature during the warmest seasons (summer and autumn) had a tremendous influence on developing ketosis in Holstein cows in a hot environment [41]. Nowadays, an increasing number of farmers rely on sophisticated sensor technologies for continuous and automated real-time monitoring of animal behaviours and, in a way, their health status [42].

## 4. Conclusions

According to the results of our study, we can conclude that subclinical ketosis has a strong relation with locomotion behaviour. Cows with SCK were more prone to change their activity (between ruminating, eating and drinking) more frequently. Lower minimal and maximal ambient temperature show a connection with the onset of SCK. Future studies with a larger number of cows are needed to confirm these results.

## Figures and Tables

**Table 1:** Definition of the activity and ambient temperature characteristics registered by RumiWatch System (Itin+Hoch GmbH, Liestal, Switzerland).

Characteristic	Definition
Walking activity (Act)	Sum of the duration of all walking bouts presented as minutes within a given recording period
up time (UT)	Time spent feeding with the head positioned upwards (min/h)
down time (DT)	Time spent feeding with the head positioned downwards (min/h)
average temperature (AT)	Average ambient temperature (°C)
minimal temperature (MinT)	Minimal ambient temperature (°C)
maximal temperature (MaxT)	Maximal ambient temperature (°C)
activity change (ActCh)	Number of times switched between activities (between other activity, ruminating, eating and drinking)

**Table 2:** Descriptive statistic of activity indicators by groups of cows.

Indicator	SCK Group		Healthy Group		*p*
	M	SE	M	SE	
Walking activity (Act)	64.33	2.558	70.46	3.261	0.140
Up time (UT)	27.45	1.496	31.29	1.707	0.092
Down time (DT)	28.11	1.243	27.51	1.129	0.719
Average temperature (AT)	19.69	0.364	18.85	0.353	0.098
Minimal temperature (MinT)	31.4	0.598	36.43	0.491	<0.003 *
Maximal temperature (MaxT)	34.29	0.508	37.69	0.402	0.001 *
Activity change (ActCh)	11.55	0.239	7.54	0.251	<0.001 *

* = significant differences. M—Means. SE—standard errors.
